# Modeling the effect of observational social learning on parental decision-making for childhood vaccination and diseases spread over household networks

**DOI:** 10.3389/fepid.2023.1177752

**Published:** 2024-01-12

**Authors:** Tamer Oraby, Andras Balogh

**Affiliations:** School of Mathematical and Statistical Sciences, The University of Texas Rio Grande Valley, Edinburg, TX, United States

**Keywords:** social learning, children vaccination, networks, disease model, social norms, Bayesian aggregation

## Abstract

In this paper, we introduce a novel model for parental decision-making about vaccinations against a childhood disease that spreads through a contact network. This model considers a bilayer network comprising two overlapping networks, which are either Erdős–Rényi (random) networks or Barabási–Albert networks. The model also employs a Bayesian aggregation rule for observational social learning on a social network. This new model encompasses other decision models, such as voting and DeGroot models, as special cases. Using our model, we demonstrate how certain levels of social learning about vaccination preferences can converge opinions, influencing vaccine uptake and ultimately disease spread. In addition, we explore how two different cultures of social learning affect the establishment of social norms of vaccination and the uptake of vaccines. In every scenario, the interplay between the dynamics of observational social learning and disease spread is influenced by the network’s topology, along with vaccine safety and availability.

## 1 Introduction

Human herding behavior, or cascading, is a convergence of opinions driven by social learning (1, 2). Such herding could also be induced by factors such as payoff externalities, sanctions, preference interaction, direct communication, and observational influence (2). Observational influence involves integrating learned behavior or the perception of others’ opinions into one’s own opinion. This leads to observational social learning, exerting a subtle pressure on people to conform (3–5). Social learning occurs through various channels of information exchange, including observation and perception of public choices (6). However, boundedly rational observational learning occurs in the presence of incomplete or insufficient information on the behavior of others (7).

Social learning has been shown to play a vital role in decision-making by people, even in the presence of information (8). Parents learn about the vaccination choices and attitudes of other parents and look for consensus as signals for vaccination decisions (9). When parents openly share their opinions about vaccinating their children, it can exert pressure on other parents (10, 11). Mixed messages and varied vaccination choices of parents can cause interpretive difficulties for others. Incomplete information about vaccines, combined with the opinions of friends on social networks, creates “boundedly rational social agents.” This situation can lead to challenges in vaccine acceptance, which can subsequently affect vaccine uptake and the spread of diseases (12).

People and households are interconnected through various types of networks. From the perspective of graph theory, there are several network models, such as the Erdős–Rényi (random) network (ERN) model and the Barabási–Albert network (BAN) model (13). These different models represent a range of real-life systems. Many real-life networks are scale-free networks (SFN), characterized by a degree distribution that follows a power law with an exponent in the range 2<γ≤3 (14). Furthermore, the structure of the network plays a crucial role in determining the effectiveness of vaccination strategies (15, 16).

Many studies have modeled the spread of diseases on networks (17–20) and on multiplex network models (21–26) Some have also considered the structure of the home in their models (27–30). However, fewer models have explored the spread of diseases and vaccine decision-making on these networks through approaches like the voter model (25), DeGroot’s selection model (14), social norms (31), and social learning (32). Moreover, most mathematical models assume rational agents making vaccination decisions with complete information (10, 17, 33, 34) with a few exceptions like Wang et al. (26).

In this study, we consider bilayer networks, focusing on two overlapping networks of the same type and their mutual influence. The first network is a physical network, where face-to-face contacts and pediatric disease transmission occur. The second network is a social one—bidirectional, weighted network through which information and opinions about vaccines are shared, influencing parents’ decisions. A pediatric disease spreads through the physical network, both within and between households, while information, opinion, and perceptions are exchanged on the social network, primarily among parents. The two networks overlap significantly, with parents connected to a wide array of other parents, relatives, coworkers, and friends. However, not all these connections necessarily exist in the physical network of children. We introduce adjustments to the network models to better reflect key characteristics of household networks. First, our model accounts for the number of children in each household. Second, households without children do not have connections in the physical network. Essentially, the expected degree of a household in the network correlates with the number of children who reside there.

In this paper, we introduce a new model focused on parental decision-making to protect children from a measles-like disease spreading through household networks. Our model incorporates boundedly rational observational social learning, utilizing a Bayesian aggregation formula distinct from the quasi-Bayesian model of boundedly rational observational learning in a general context, as presented by Mueller-Frank and Neri (7). We demonstrate that our model not only gives rise to social norms but also encompasses other selection models, including voting and DeGroot’s models. In our approach, we consider socially bounded agents—specifically, parents who promote their children’s wellbeing—who have imperfect information regarding the vaccination choices of their neighbors in the network.

We hypothesize that these agents are only capable of perceiving messages as either correct or incorrect, possibly due to fear of retribution or confusion. There is a likelihood that agents will communicate an accurate representation of their opinion with a probability of q, and conversely, there is a probability of 1−q for conveying an inaccurate message [see, e.g., Easley and Kleinberg (35)]. Utilizing this model, we explore the cascading effect of opinions on vaccination within the context of boundedly rational observational social learning, and we compare our findings to other models of social pressure [see Phillips et al. (31) and Oraby et al. (36); also see, e.g., Oraby and Bauch (12) and Ortega and Braun (37)]. Furthermore, we examine the impact of such signal games on the propagation of vaccine-related opinion and diseases across social and physical networks, especially in scenarios with limited resources reflected in vaccine efficacy and accessibility, and safety. Finally, our study investigates how the presence of two different cultures of social learning influences the establishment of a social norm and, consequently, vaccine uptake.

## 2 Model and methods

### 2.1 Networks

To model the spread of the disease, we employ an agent-based network model N households serve as nodes. Each household contains a certain number of children, denoted as $C_{i}$ (with $0\leq C_{i}\leq n_{C}$, for i=1,…,N), who are interconnected through a physical network. Here, $n_{C}$ represents the maximum number of children per household. Parents are interconnected through a separate bidirectional weighted network—encompassing social, Internet, and physical connections—through which they exchange opinions, share information, and observe choices. Notably, parents in childless households can still be connected to other parents and influence their opinions. Our model utilizes two types of networks: the ERN model and the BAN model (13). In the physical network, we hypothesize that the degree of nodes is proportional to the number of children in each household. Meanwhile, the parent network overlaps with the children’s network through random rewiring, with the probabilities of forming new connections being higher than those for severing existing ones. The parent network is assumed to be a weighted bidirectional network, with weights represented by learning probabilities $q_{\;j,i}$, for each social network link (i,j), where i,j=1,…,N. For comprehensive details about the network models, refer to the Supplementary Material (SI, Model). Figure S1 in the Supplementary Material illustrates a histogram for each type of random network.

### 2.2 Birth process

We postulate a birth process that depends on the number of children already residing in a household. The probability of a new pregnancy is modeled using a logistic function with a median value of $C^{*}$ and decreasing as the household’s number of children approaches $n_{C}$. For detailed information, refer to the Supplementary Material (SI, Model). The gestation period for a pregnancy is set at 280 days. Upon the birth of a newborn, while the household’s child count is updated, the number of connections in the children’s network remains unchanged. Due to their rarity, miscarriages and child mortality are not included in our model.

### 2.3 Disease spread

We assume the spread of a new measles-like disease, which is vaccine-preventable and affects only children. This disease spreads within and between households. The incubation period has a mean duration of $m_{p}$ days, with a maximum of ℓ days. The probability of a new infection occurring in a household is given by $1-(1-\beta_{h}{)}^{I(i)}\cdot (1-\beta {)}^{n_{I}(i)/C_{i}}$, where β represents the probability of infecting a child in a different household (through the physical network) and $\beta_{h}$ is the probability of infecting a sibling within the same household. Here, I(i) denotes the total number of infected siblings in the same household, while $n_{I}(i)$ is the number of infected children connected to the household i through the network. The number $n_{I}(i)/C_{i}$ is used to approximate the probability of infection, based on the assumption that children in a household, on average, have an equal number of friends. We assume that the epidemic begins with $I_{0}$ initially infected children randomly distributed across different households.

### 2.4 Vaccination decision-making

Parents are classified into three groups: “never-vaccinators,” who consistently oppose vaccination; “non-vaccinators,” who may or may not choose to vaccinate; and “vaccinators,” who are inclined to vaccinate. A small percentage of parents are “never-vaccinators,” yet they continue to share their opinions. In household i, parents make their decision about vaccinating their children based on the perceived reward of vaccination, calculated as $\pi_{i}=\alpha_{i}I-\gamma_{i}A$. Here, I represents the total number of infected children and A denotes the total number of adverse events related to the vaccine, both considered up to the point of decision-making. Parents who experience an adverse event after vaccinating their children subsequently become “never-vaccinators.” The parameter $\alpha_{i}$ indicates the importance of the infectiousness of the disease (degree of relevance), and $\gamma_{i}$ reflects the importance (degree of relevance) of vaccine adverse events in shaping the subjective opinion of the family i. The probability of the household i accepting vaccination is given by $pr_{i}=1/(1+\exp(-\pi_{i}))$, except for “never-vaccinators,” for whom this probability is zero.

We assume that on day 1 of the epidemic, the vaccine would not have been used, and therefore no adverse events would have occurred. Thus, the initial stance of parents in the household i toward vaccinating their children will be determined solely on the perceived severity of the disease, that is, using probabilities $pr_{i}=1/(1+\exp(-\alpha_{i}I_{0}))$.

### 2.5 Observational social learning

Let $q_{i,j}$ represent the learning probability that household i correctly perceives or learns about household j’s opinion or position on vaccination. On the contrary, $1-q_{i,j}$ denotes the probability that the household i misinterprets the opinion of the household j. This social learning is not necessarily symmetric, that is, $q_{i,j}$ may not equal $q_{\;j,i}$. For example, followers of a celebrity tend to learn more from the celebrity than vice versa. n cases of reciprocal or symmetric social learning, $q_{\;j,i}=q_{i,j}$. Let us consider that the household i has a set of vaccinator neighbors in the social network, denoted as $N_{V}(i)$, with cardinality $n_{V}(i)$, and a set of non-vaccinator neighbors, $N_{N}(i)$, with cardinality $n_{N}(i)$. In addition, the total number of neighbors is given by $n_{S}(i)=n_{V}(i)+n_{N}(i)$, where $N_{S}(i)=N_{V}(i)\cup N_{N}(i)$. Then parents in household i make the decision to vaccinate their children based on the following posterior probability:

(1) $$P_{S}(i)=\frac{\;pr_{i}\cdot \prod_{\;j\in N_{V}(i)}q_{\;j,i}\cdot \prod_{k\in N_{N}(i)}(1-q_{k,i})}{\begin{array}{c} pr_{i}\cdot \prod_{\;j\in N_{V}(i)}q_{\;j,i}\cdot \prod_{k\in N_{N}(i)}(1-q_{k,i})\;\\ +\;(1-pr_{i})\cdot \prod_{\;j\in N_{V}(i)}(1-q_{\;j,i})\cdot \prod_{k\in N_{N}(i)}q_{k,i} \end{array}}.$$

This is called Bayesian aggregation rule in observational social learning. The rationale behind (1) is as follows: the prior probability of vaccination, $pr_{i}$, is updated based on independent information collected or perceived from neighbors in the network. A vaccinator is perceived to hold his/her opinion with a probability of $q_{i,j}$, while a non-vaccinator is perceived to favor vaccination with probability $1-q_{i,j}$. For more detailed information on the posterior probability described in Equation (1) and its connections to other models in the literature, such as the voting model and the DeGroot model, please refer to the Supplementary Material titled “SI, Model.”

Each day, parents’ positions on vaccination are updated randomly based on probabilities $P_{S}$. In addition, parents who are vaccinators are selected with a probability ρ to vaccinate all their children. This probability ρ represents the probability of gaining access to vaccination, which depends on the available resources.

### 2.6 Epidemiological measures

To analyze the impact of learning probability $q_{\;j,i}$ on parents’ opinions and disease spread, we use several epidemiological measures: the size of the epidemic, the epidemic peak, the uptake of vaccines, the number of vaccinators, and the basic reproduction number $R_{0}$. We specifically use $R_{0}$ for calibration the ERN, treating it as an epidemiological measure rather than a threshold (38). The size of the epidemic is defined as the total number of children infected by the end of the epidemic, while the uptake of vaccines refers to the total number of vaccinated children. The final number of vaccinators is considered to assess whether a consensus on vaccination emerges by the end of the epidemic. The basic reproduction number $R_{0}$ is defined as the average number of secondary cases in a completely susceptible population. This definition is used to develop an algorithm to estimate the value of $R_{0}$ (see Algorithm 1 in Supplementary Material SIII, Methods). This algorithm applies Bayes’ theorem to determine the probability of infection from contact with the index case, which aids in calculating the average number of infections. To determine the overall average, we first average the results over multiple disease transmission simulations, then across the N households (which may include the index case), and finally over simulations of various randomly generated networks.

### 2.7 Model simulation

The model is implemented using stochastic simulation of 100 runs to investigate the impact of the social learning probability $q_{\;j,i}$ on vaccine uptake and the spread of pediatric diseases. We set $q_{\;j,i}$ to be uniformly distributed within the range q±0.05 for a pre-specified value of q, where 0<q<1. At the beginning of each time step (day) we update the vaccination stance and the disease states of the infected children. In the network, multiple infections can occur on the same day, and the numbers $n_{I}(i)$ and I(i) are updated daily for all households i, where i=1,2,…,N. An infected child on the jth day post-infection either recovers, making the end of the incubation period, or remains infected, with the transition to the next day. These transitions are determined by probabilities calculated from a truncated exponential distribution. For further details, please refer to the Supplementary Material SI, Model.

Our simulation codes utilize the NumPy-compatible CuPy Python library (39), which is accelerated using NVIDIA CUDA (40) for parallel computations on graphical processing units (GPUs). Most of our calculations were conducted on a GPU cluster equipped with eight NVIDIA Tesla (Kepler) K80 GPU cards; each card boasts 2,496 CUDA Cores and 12 GB of memory. Furthermore, the code was tested on a GPU cluster comprising eight NVIDIA A100 SXM GPU cards, each featuring 6,912 CUDA cores and 80 GB of memory. For further details, please refer to the Supplementary Material SV, Coding of Simulations.

### 2.8 Parameter values

We have parameterized our model using reviews of the literature, calibration, and guesstimation. The model includes N=100,000 households, representing a medium-sized city. Each household randomly contains an average of two and a half children. In the ERN, the average degree is assumed to be 40 for the children’s network and 60 for the parents’ network. The epidemic is postulated to begin with $I_{0}=10$ initially infected children, randomly dispersed among the N households. The disease is modeled with an average incubation period of 11 days and a maximum of 16 days [see Vynnycky and White (41, p. 8)]. We calibrated the value of β using values of $R_{0}$ ranging from 12 to 18 (41, p. 8). Furthermore, we assume that 5% of the population, for medical or ideological reasons, will categorically refuse vaccination (never-vaccinators). A comprehensive table detailing the definitions of parameters and their respective values is available in Supplementary Material SII, Table S1.

## 3 Results

**Figure 1:**
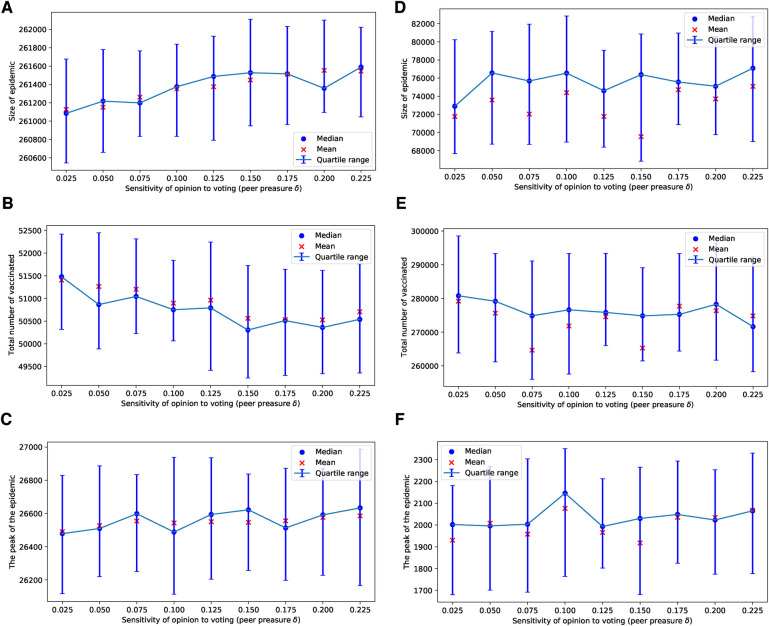
Simulations of sizes of the epidemic, the total number of vaccinated children, and the peak of the epidemic for different values of δ. Simulations are done on the ERN model in (**A**–**C**) and on the BAN model in (**D**–**F**). In all simulations, $P_{adv}=0.0001$ and ρ=0.01.

In our use of voting models of selection, we observed that the impact of the degree of injunctive social norm or peer pressure, denoted by δ, on epidemic sizes, their peaks, and vaccine uptake is almost negligible for selected values of δ in the range of [0.025,0.225], as shown in Figure 1. This effect is particularly subtle for the ERN model as demonstrated in Figures 1A–C. It is more pronounced in the Barabási–Albert network model, as seen in Figures 1D–F. The degree of injunctive social norm influences vaccine uptake and the sizes and peaks of epidemics differently in these two types of networks, as indicated by the contrasting results in the left and right panels of Figure 1. Notably, vaccine uptake in Barabási–Albert networks remains higher compared to Erdős–Rényi networks, regardless of group pressure.

**Figure 2:**
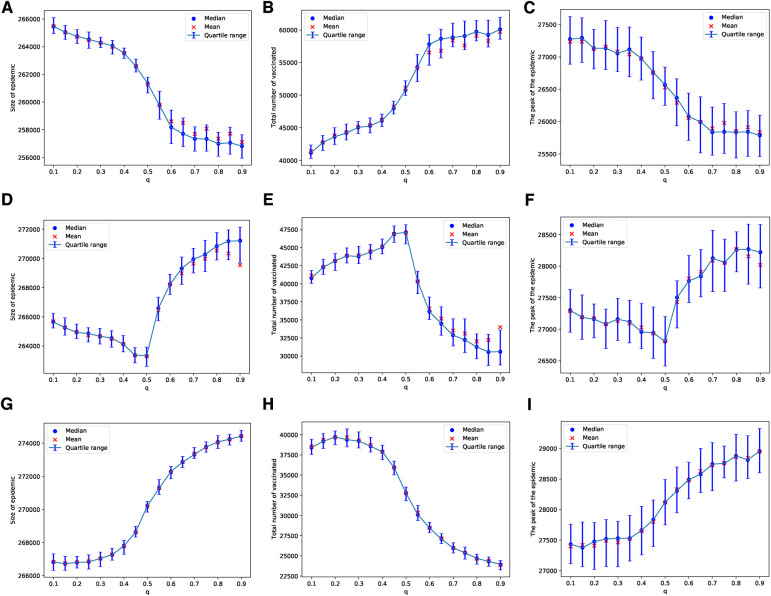
Simulations of sizes of the epidemic, the total number of vaccinated children, and the peak of the epidemic on the ERN model for different values of q. Simulations are done using $P_{adv}=0.0001$ in (**A**–**C**), $P_{adv}=0.001$ in (**D**–**F**), and $P_{adv}=0.01$ in (**G**–**I**). In all simulations, ρ=0.01.

In the case of the ERN model, and using the general Bayesian aggregation rule as outlined in Equation (1), we observe more complex dynamical behaviors compared to those resulting from the voter model with an injunctive social norm or peer pressure δ. First, we assume that there are enough vaccines to vaccinate 1 out of every 100 children daily. When $P_{adv}=0.0001$, an increase in q intensifies the pressure on parents, leading to a higher vaccine uptake and, consequently, to smaller epidemic sizes and peaks, as shown in Figures 2A–C. However, as the probability of an adverse event increases, resulting in more adverse events, we find that an increase in the correct perception probability q correlates with a decline in vaccine uptake. Specifically, with $P_{adv}=0.001$, vaccine uptake continues to rise with values of q<0.5, but this pattern shifts for q>0.5, as illustrated in Figures 2D–F. At a higher adverse event probability of $P_{adv}=0.01$, vaccine uptake decreases as the value of q increases beyond approximately q∼0.2. In this scenario, both the size and the peak of the epidemic grow with an increase in the probability q, as depicted in Figures 2G–I.

**Figure 3:**
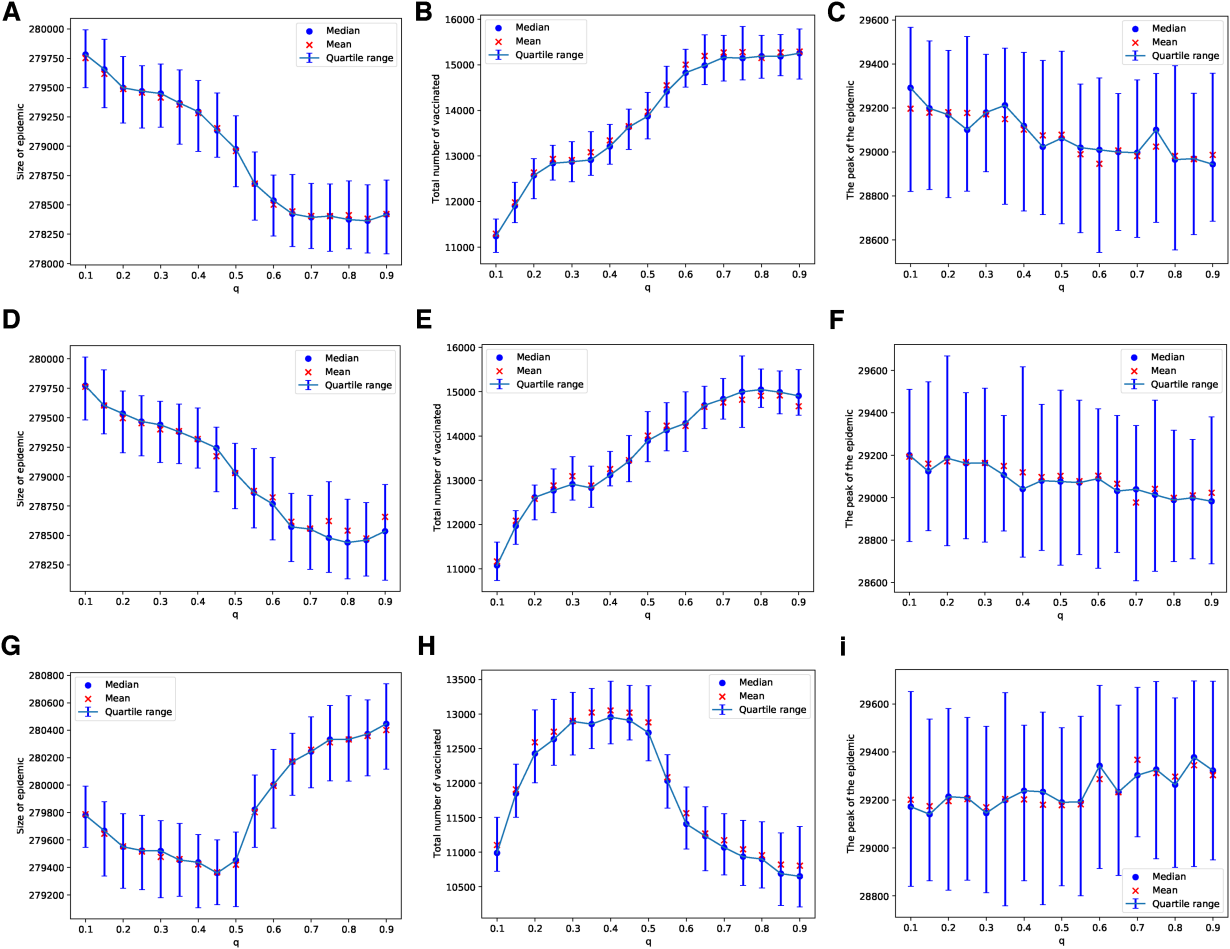
Simulations of sizes of the epidemic, the total number of vaccinated children, and the peak of the epidemic on the ERN model for different values of q. Simulations are done using $P_{adv}=.0001$ in (**A**–**C**), $P_{adv}=0.001$ in (**D**–**F**), and $P_{adv}=0.01$ in (**G**–**I**). In all simulations, ρ=0.001.

These patterns change when we consider a scenario with limited vaccine availability, such as the ability to vaccinate only 1 out of every 1,000 children each day. In this case, the uncertainty of the results increases; this can be observed by comparing the panels in Figure 2 with those in Figure 3. The lower the availability of vaccines, the fewer the number of adverse cases that occur. A higher probability of an adverse event is required to effectively discourage parents from vaccinating their children, as depicted in Figures 3B,E,H. In particular, under these conditions, the peaks of epidemics do not show significant changes with variations in the probability of learning.

**Figure 4:**
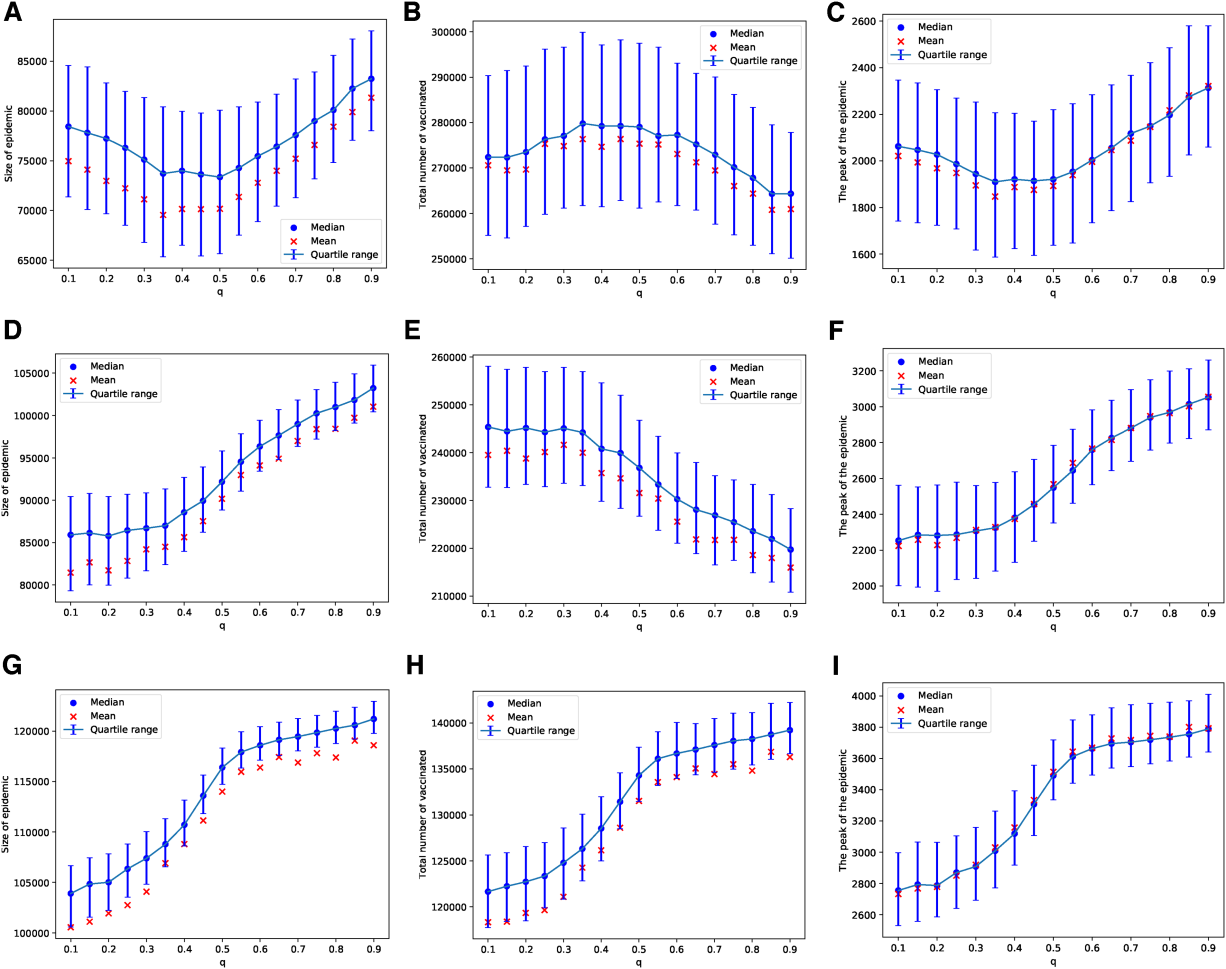
Simulations of sizes of the epidemic, the total number of vaccinated children, and the peak of the epidemic on the BAN model for different values of q. Simulations are done using $P_{adv}=0.0001$ in (**A**–**C**), $P_{adv}=0.001$ in (**D**–**F**), and $P_{adv}=0.01$ in (**G**–**I**). In all simulations, ρ=0.01.

**Figure 5:**
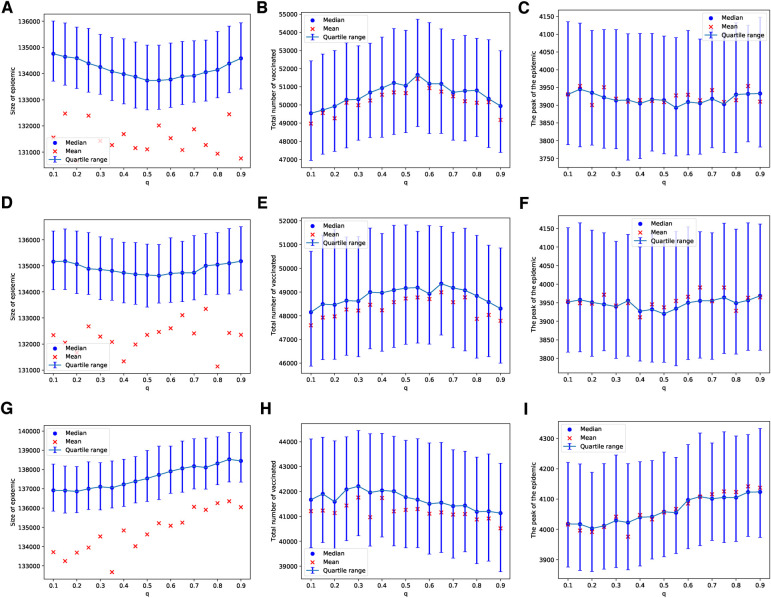
Simulations of sizes of the epidemic, the total number of vaccinated children, and the peak of the epidemic on BAN model for different values of q. Simulations are done using $P_{adv}=0.0001$ in (**A**–**C**), $P_{adv}=0.001$ in (**D**–**F**), and $P_{adv}=0.01$ in (**G**–**I**). In all simulations, ρ=0.001.

In the case of the BAN model and using the Bayesian update rule given in Equation (1), we find that the epidemic sizes are smaller compared to those in ERNs. This observation can be made by comparing the panels of Figure 2 with Figure 4, and the panels of Figure 3 with Figure 5. This finding contradicts the well-known fact that diseases tend to spread faster in BANs. However, this discrepancy could be attributed to the increased vaccine uptake observed in BANs compared to ERNs. This difference between BANs and ERNs is also evident in the context of different values of q. For example, when the probability of an adverse event increases to $P_{adv}=0.01$, the levels of vaccine uptake drop from those observed at $P_{adv}=0.0001$ and $P_{adv}=0.001$. This can be seen by comparing panel (H) of Figure 4 with panels (B) and (E). However, in this scenario, the probability of learning exerts pressure to increase vaccine uptake, even if it leads to more adverse events, as illustrated by comparing panel (H) of Figure 4 with panel (H) of Figure 2.

Similarly, these patterns become less pronounced when we consider a scenario where vaccines are scarce, being available for only 1 in every 1,000 children each day. As with the ERNs, this assumption leads to increased outcome uncertainty, which can be observed by comparing the panels of Figure 4 with Figure 5. However, in contrast to the ERNs, the learning probability in the BANs does not significantly influence vaccine uptake, nor does it affect the size and peak of the epidemics. This can be seen by comparing panels of Figure 3 with those of Figure 5.

**Figure 6:**
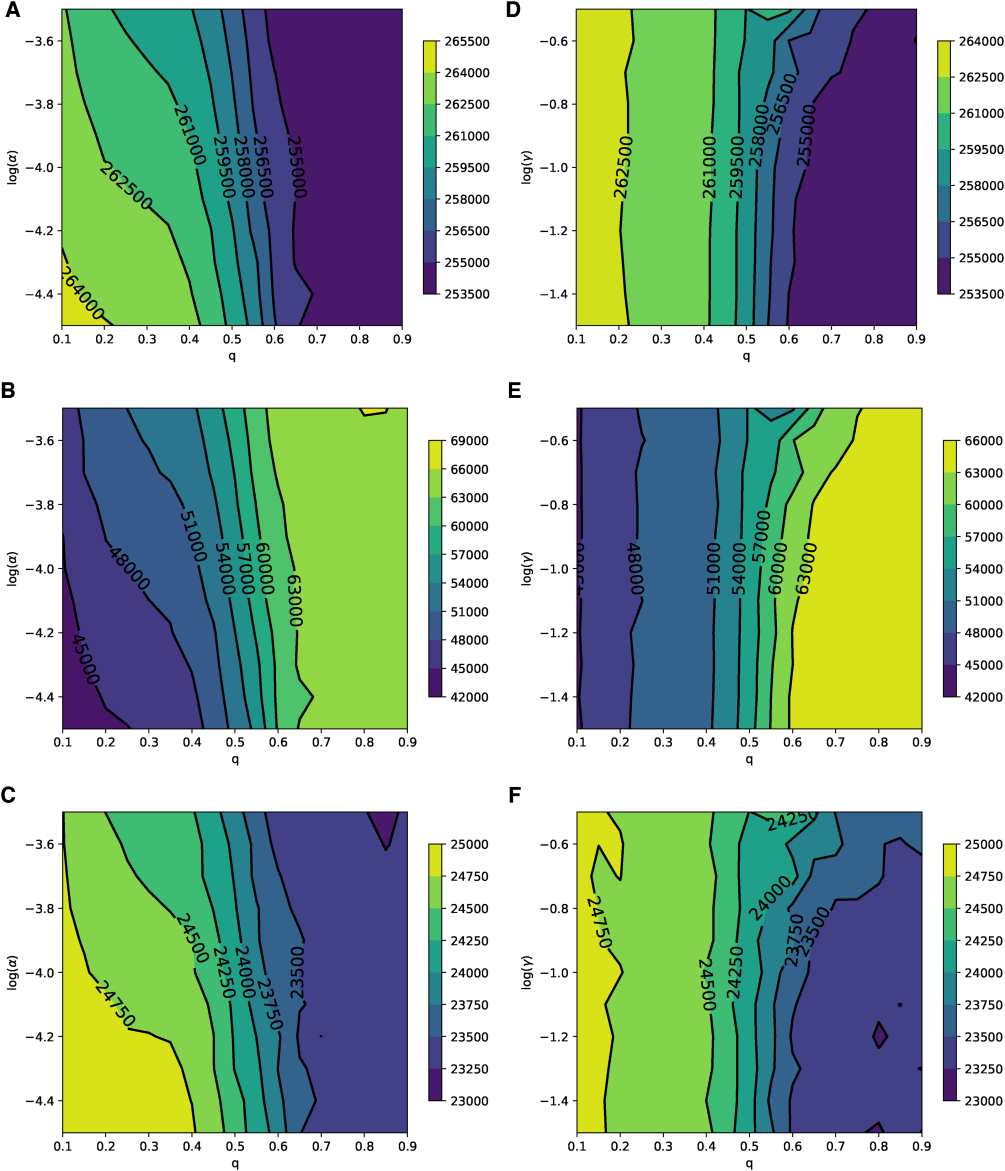
Parameter planes of *α* against *q* in (**A–C**) and *γ* against *q* in (**D–F**) for epidemic sizes, the total number of vaccinated children, and the peak of the epidemic on the ERN model. In all simulations, the median value of the simulations is used to plot the parameter planes that are performed at $P_{adv}=0.0001$ and ρ=0.01.

The parameter planes depicted in Figure 6, which represent the relevance of the disease (α) and the relevance of the adverse event of the vaccine (γ) to the rational choice component for different values of q, show patterns consistent with the simulation results presented in Figure 2. This indicates that in ERNs, the effect of learning significantly influences (suppresses) the rational perception of parents regarding the benefits of vaccination. The parameter planes in Supplementary Figures S2 and S3 also show consistent patterns.

**Figure 7:**
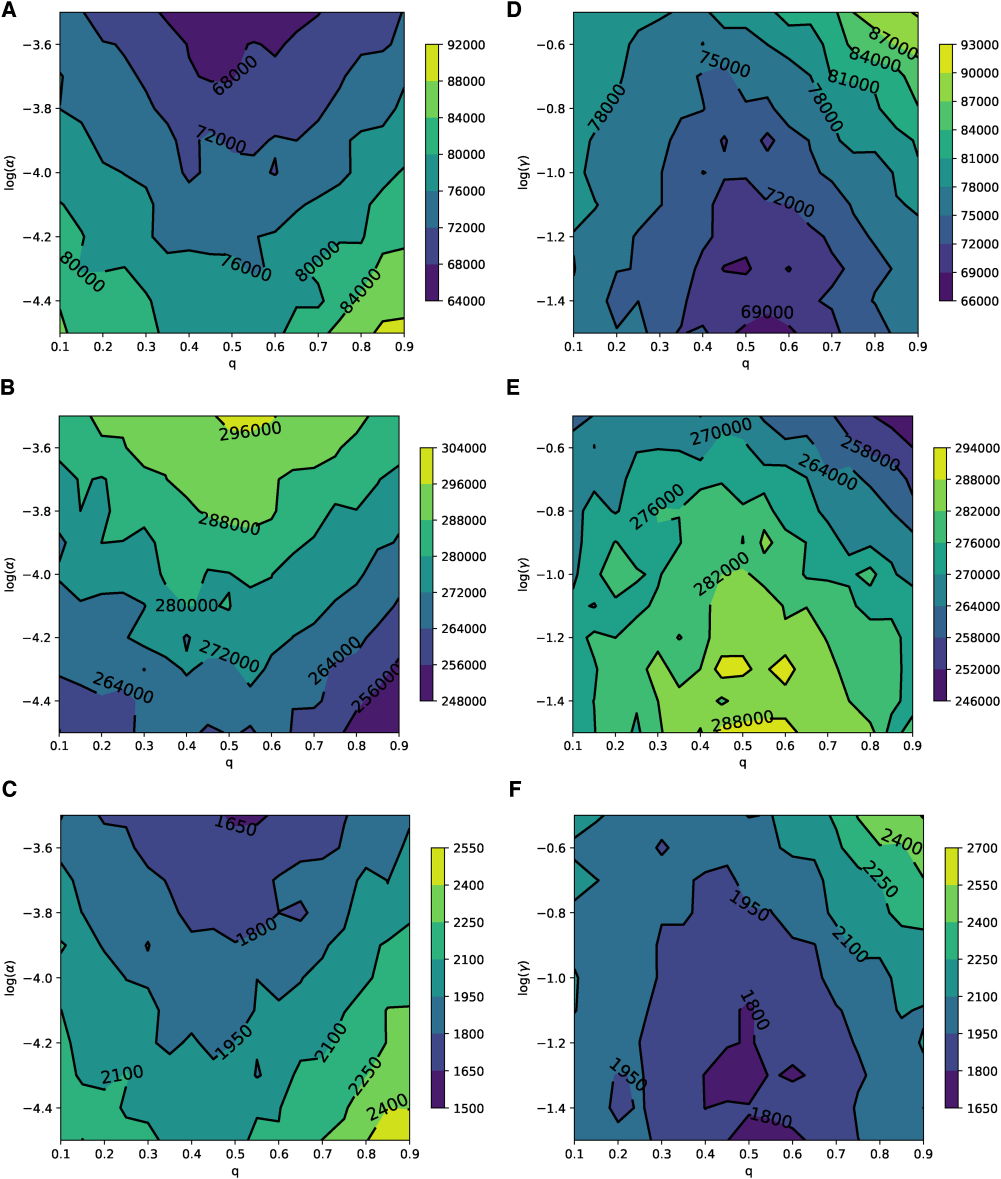
Parameter planes of *α* against *q* in (**A–C**) and *γ* against *q* in (**D–F**) for epidemic sizes, the total number of vaccinated children, and the peak of the epidemic on the BAN model. In all simulations, the median value of the simulations is used to plot the parameter planes that are performed in $P_{adv}=0.0001$ and ρ=0.01.

In Barabási–Albert networks, similar to Erdős–Rényi networks, the parameter planes shown in Figure 7 illustrate the relevance of the disease (α) and the vaccine’s adverse event (γ) to the rational choice component for different values of q. These planes exhibit patterns that are consistent with the simulations in Figure 4. Qualitatively, in BANs, both diseases and opinions spread in a way where vaccine uptake and epidemic responses can be symmetric to both high and low learning probabilities. Quantitatively, the rational perception of parents regarding the payoff of vaccination influences vaccine uptake and epidemic dynamics. As the perceived risk of the disease increases, vaccine uptake also increases, leading to reduced epidemic sizes and peaks, as shown in Figures 7A–C. Conversely, as the perceived risk of adverse events from vaccine increases, vaccine uptake decreases, which in turn leads to larger epidemic sizes and peaks, as depicted in Figures 7D–F. The parameter planes in Supplementary Figures S4 and S5 also show similar consistent patterns.

**Figure 8:**
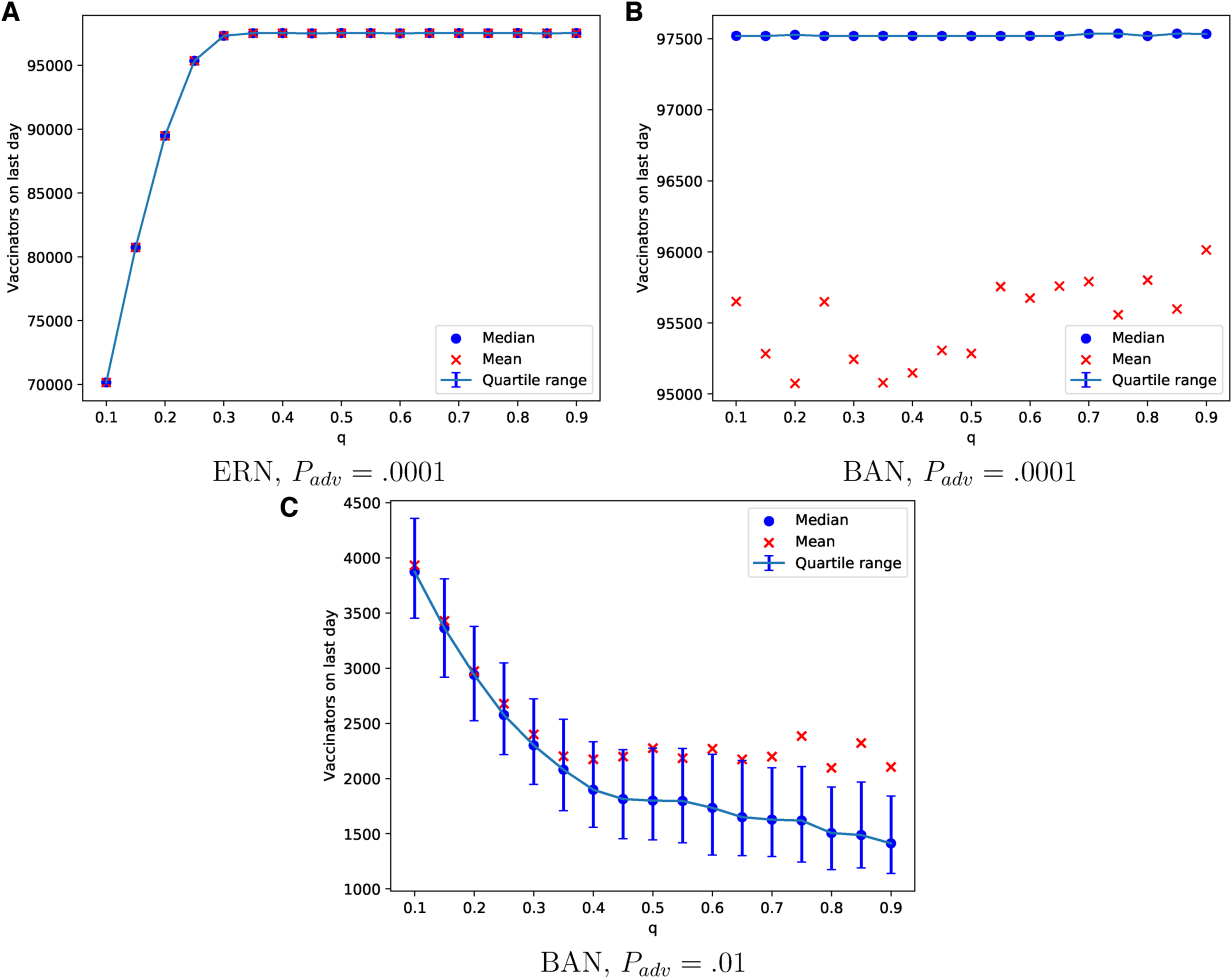
Simulations of the final total number of vaccinators on ERN model when *P*_adv_ = .0001 in (**A**), BAN model when *P*_adv_ = .0001 in (**B**), and BAN model when *P*_adv_ = .01 in (**C**) for different values of q.

The probability of learning from parents can lead to a consensus on vaccination in both ERN and BAN models, as shown in Figures 8A,B. This is evident in the number of vaccinators on the last day of the epidemic. However, achieving this consensus in the ERN typically requires a moderate to high learning probability. The pattern in ERNs remains consistent with that in Figure 8A even when $P_{adv}=0.01$, though these data are not shown to avoid redundancy. On the contrary, the pattern changes significantly for BANs under the same conditions ($P_{adv}=0.01$), as shown in Figure 8C. In this scenario, fewer parents in BANs choose to vaccinate their children, and acceptance of the vaccine declines as the probability of learning increases.

Until this point, our model simulations have assumed a homogeneous culture within the population. Specifically, parents reveal their actual preference or strategy based on probabilities $q_{i,j}$ within the range of (q−0.05,q+0.05) for a fixed value of q. In the next part of our study, we explore the impact of introducing a cultural attribute to the population. This addition results in a heterogeneous population, divided into two groups, each employing probabilities within two distinct ranges: 0.1±0.05 and 0.9±0.05. We have selected 0.1 and 0.9 to represent extreme cases. The group associated with the latter range is referred to as the subpopulation with attribute 1. The proportion of individuals within this subpopulation can significantly influence the outcomes of vaccine uptake and the spread of the epidemic.

**Figure 9:**
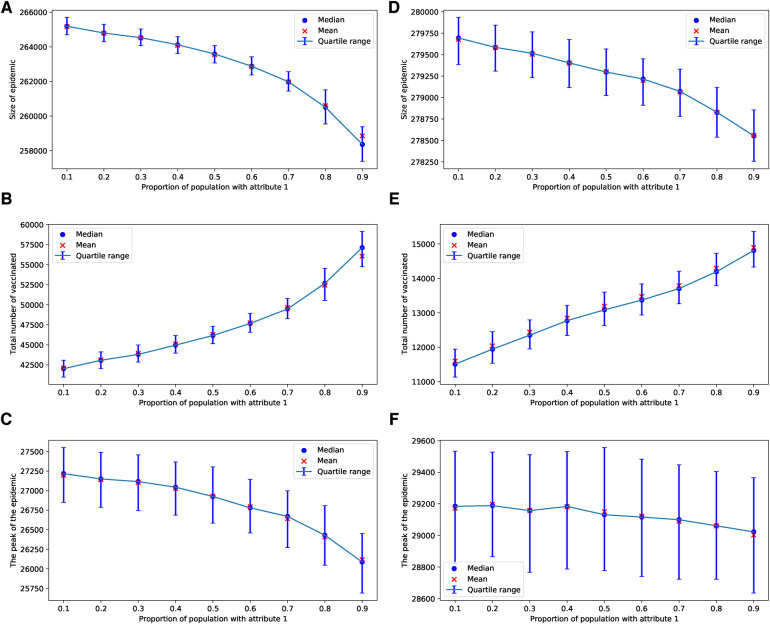
Simulations of sizes of epidemic, total number of vaccinated children, and the peak of the epidemic on the ERN model for different proportions of attribute 1. Simulations are done using ρ=0.01 in (**A**–**C**), and ρ=0.001 in (**D**–**F**). In all simulations, $P_{adv}=0.0001$.

**Figure 10:**
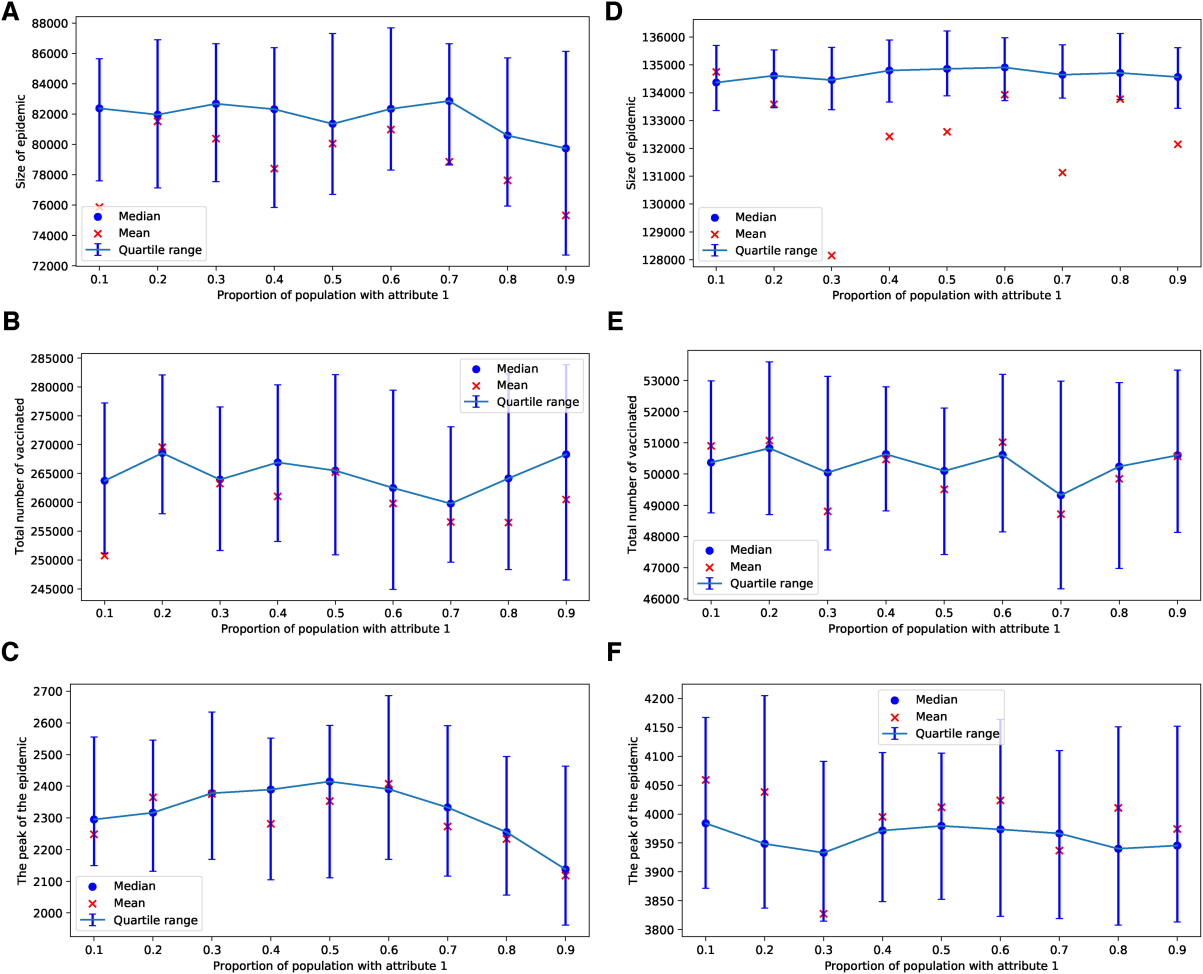
Simulations of sizes of the epidemic, the total number of vaccinated children, and the peak of the epidemic on BAN model for different proportions of attribute 1. Simulations are done using ρ=0.01 in (**A**–**C**) and ρ=0.001 in (**D**–**F**). In all simulations, $P_{adv}=0.0001$.

In the case of an epidemic spreading through an ERN, a small proportion of parents with attribute 1 leads to a larger epidemic size and peak, as well as lower uptake of the vaccine. This outcome is evident in the simulation results presented in Figure 9. In contrast, when an epidemic spreads through a BAN, the heterogeneity in the population culture does not significantly affect the dynamics of the epidemic or the uptake of the vaccine, as shown in the simulation results in Figure 10.

**Figure 11:**
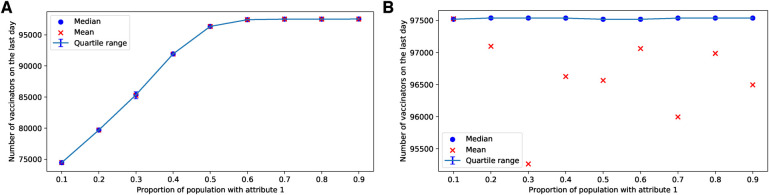
Simulations of final total number of vaccinators on ERN and BAN models for different proportions of attribute 1. Simulations are done on the ERN model in (**A**) and the BAN model in (**B**). All the simulations are done using $P_{adv}=0.0001$ and ρ=0.01

If more than half of the parents possess the attribute 1, the parents reach a consensus to vaccinate in the case of the ERNs, as shown in Figure 11A. In the case of BANs, vaccination becomes a consensus in all scenarios, as depicted in Figure 11B.

## 4 Discussions and conclusion

In this paper, we introduce a Bayesian aggregation model for boundedly rational observational social learning, focusing on decision-making process related to vaccination. This learning model is based on social observations of neighbors within a social network, where information dissemination occurs. We demonstrate that other models in the literature are special cases of our model. However, some models, such as those based on the degree of injunctive social norm (δ), do not offer the scalability that our model provides. Using our new model, we investigate how social learning influences the development of consensus on a network and examine the impact of cultural heterogeneity in observational social learning on vaccine uptake. Our approach involves stochastic simulations of disease and information spread across two overlapping networks, specifically the ERN model and the BAN model. The results of observational social learning on these networks and their mutual influence on disease spread within the overlapping network varied depending on the type of network, as well as vaccine safety and availability. In ERNs, an increase in learning pressure, particularly when q≥0.5 (the rational agent case), leads to increased vaccine uptake and the potential establishment of vaccination as a social norm, especially when adverse events are rare or vaccines are inaccessible. The uptake of vaccines in BANs is generally higher than in ERNs, whereas epidemic sizes are smaller. However, in BANs, a higher learning probability (q≥0.5) results in a reduced uptake of vaccine.

Our simulations of epidemic processes on ERNs and BANs indicate that degree distribution plays a significant role in vaccine uptake levels and parental acceptance of vaccination. In the case of BANs, vaccine uptake is higher compared to ERNs, which can be attributed to two main reasons. First, since diseases are known to spread rapidly in BANs, the rapid accumulation of cases increases the subjective probability of opting for vaccination. Second, the greater number of neighbors in BANs amplifies the tendency toward one of the two opinions, even with small values of q.

Vaccine availability and accessibility, along with the likelihood of adverse events, interact in a way that can significantly influence parental opinion and, consequently, vaccine uptake. In ERNs, increased vaccine availability coupled with lower chances of adverse events helps boost vaccine uptake and establish vaccination as a consensus. However, in BANs, this paradigm shifts due to its heavy-tailed degree distribution.

Mixed populations, featuring two different cultures of sharing and perceiving opinions about vaccination, can greatly affect vaccine uptake. In a population where a fraction has a lower learning probability ($q_{1}$) compared to the rest ($q_{2}>q_{1}$), the total uptake of vaccines may decrease and the size of the epidemic may increase, compared to a homogeneous population with a consistent learning probability ($q_{2}$).

Social studies employing surveys and behavioral game experiments should consider the personal characteristics of each parent that lead to directional learning probabilities ($q_{i,j}$ and $q_{\;j,i}$). These population surveys can be used to predict vaccine uptake levels. To effectively increase vaccine uptake, it is insufficient to only consider the degree of parental linkage in the information network to spread awareness. Enhanced efforts to promote social information exchange and social norm interventions are necessary to encourage prosocial vaccination decisions (3–5, 42). According to our model, such efforts can lead to a consensus on vaccination opinions and increase vaccine uptake, even in scenarios with a significant presence of never-vaccinators and despite challenges related to vaccine safety and availability.

## Data Availability

The original contributions presented in the study are included in the article/Supplementary Material, further inquiries can be directed to the corresponding author.

## References

[1] Dietz T Stern PC. Toward a theory of choice: socially embedded preference construction. J Socio Econ. (1995) 24(2):261–79. 10.1016/1053-5357(95)90022-5

[2] Hirshleifer D Hong Teoh S. Herd behaviour, cascading in capital markets: a review, synthesis. Eur Financ Manage. (2003) 9:25–66. 10.1111/1468-036X.00207

[3] Cai H Chen Y Fang H. Observational learning: evidence from a randomized natural field experiment. Am Econ Rev. (2009) 99(3):864–82. 10.1257/aer.99.3.864

[4] Sasaki S. Observing descriptive social norm, conformity: experimental evidence. J Behav Econ Finance. (2018) 10:81–94. 10.11167/jbef.10.81

[5] Goeschl T Kettner SE Lohse J Schwieren C. From social information to social norms: evidence from two experiments on donation behaviour. Games. (2018) 9(4):91. 10.3390/g9040091

[6] Bandura A Walters RH. Social learning theory. Vol. 1. Englewood Cliffs, NJ: Prentice Hall (1977).

[7] Mueller-Frank M Neri C. A general model of boundedly rational observational learning: theory, experiment. SSRN Electron J. (2015):SSRN.2566210. 10.2139/ssrn.2566210

[8] Bikhchandani S Hirshleifer DA Tamuz O Welch I. Information cascades, social learning. No. w28887. National Bureau of Economic Research (2021). 10.3386/w28887

[9] Damnjanović K Graeber J Ilić S Lam WY Lep Ž Morales S, et al. Parental decision-making on childhood vaccination. Front Psychol. (2018) 9:735. 10.3389/fpsyg.2018.00735 29951010 PMC6008886

[10] Brunson EK. The impact of social networks on parents’ vaccination decisions. Pediatrics. (2013) 131(5):e1397–404. 10.1542/peds.2012-2452 23589813

[11] Ebi SJ Deml MJ Jafflin K Buhl A Engel R Picker J, et al. Parents’ vaccination information seeking, satisfaction with and trust in medical providers in Switzerland: a mixed-methods study. BMJ Open. (2022) 12(2):e053267. 10.1136/bmjopen-2021-053267 35228281 PMC8886431

[12] Oraby T Bauch C. Bounded rationality alters the dynamics of paediatric immunization acceptance. Sci Rep. (2015) 5:10724. 10.1038/srep10724 26035413 PMC4451793

[13] Easley D Kleinberg J. Networks, crowds, and markets: reasoning about a highly connected world. Vol. 1. Cambridge: Cambridge University Press (2010).

[14] Namatame A Chen S-C. Agent-based modeling and network dynamics. New York: Oxford University Press (2016).

[15] Petrizzelli F Guzzi PH Mazza T. Beyond COVID-19 pandemic: topology-aware optimization of vaccination strategy for minimizing virus spreading. Comput Struct Biotechnol J. (2022) 20:2664–71. 10.1016/j.csbj.2022.05.040 35664237 PMC9135485

[16] Guzzi PH Petrizzelli F Mazza T. Disease spreading modeling, analysis: a survey. Brief Bioinformatics. (2022) 23(4):bbac230. 10.1093/bib/bbac230 35692095

[17] Ndeffo Mbah ML Liu J Bauch CT Tekel YI Medlock J Meyers LA, et al. The impact of imitation on vaccination behavior in social contact networks. PLoS Comput Biol. (2012) 8(4):e1002469. 10.1371/journal.pcbi.1002469 22511859 PMC3325186

[18] Pastor-Satorras R Vespignani A. Epidemics and immunization in scale-free networks. In: *Handbook of graphs and networks*. Weinheim Wiley-VCH Verlag GmbH & Co. KGaA (2004). p. 111–30.

[19] Boguñá M Castellano C Pastor-Satorras R. Nature of the epidemic threshold for the susceptible-infected-susceptible dynamics in networks. Phys Rev Lett. (2013) 111:068701. 10.1103/PhysRevLett.111.068701 23971619

[20] Keeling MJ Eames KT. Networks and epidemic models. J R Soc Interface. (2005) 2(4):295–307. 10.1098/rsif.2005.0051 16849187 PMC1578276

[21] Granell C Gómez S Arenas A. Dynamical interplay between awareness and epidemic spreading in multiplex networks. Phys Rev Lett. (2013) 111(12):1–5. 10.1103/PhysRevLett.111.128701 24093306

[22] Scatà M Di Stefano A Liò P La Corte A. The impact of heterogeneity and awareness in modeling epidemic spreading on multiplex networks. Sci Rep. (2016) 6(1):37105. 10.1038/srep37105 27848978 PMC5111071

[23] Pan Y Yan Z. The impact of multiple information on coupled awareness-epidemic dynamics in multiplex networks. Physica A. (2018) 491:45–54. 10.1016/j.physa.2017.08.082 29960396

[24] Guo Q Jiang X Lei Y Li M Ma Y Zheng Z. Two-stage effects of awareness cascade on epidemic spreading in multiplex networks. Phys Rev E. (2015) 91(1):012822. 10.1103/PhysRevE.91.012822 25679671

[25] Fátima V-R Vazquez F. Interacting opinion and disease dynamics in multiplex networks: discontinuous phase transition and nonmonotonic consensus times. Phys Rev E. (2017) 95:52315. 10.1103/PhysRevE.95.052315 PMC7219934 28618582

[26] Wang Z Andrews MA Wu ZX Wang L Bauch CT. Coupled disease-behavior dynamics on complex networks: a review. Phys Life Rev. (2015) 15:1–29. 10.1016/j.plrev.2015.07.006 26211717 PMC7105224

[27] Liu J Wu J Yang ZR. The spread of infectious disease on complex networks with household-structure. Physica A. (2004) 341(1-4):273–80. 10.1016/j.physa.2004.05.031

[28] Ball F Sirl D Trapman P. Analysis of a stochastic SIR epidemic on a random network incorporating household structure. Math Biosci. (2010) 224(2):53–73. 10.1016/j.mbs.2009.12.003 20005881

[29] Ma J van den Driessche P Willeboordse FH. Effective degree household network disease model. J Math Biol. (2013) 66(1–2):75–94. 10.1007/s00285-011-0502-9 22252505

[30] Pellis L Ball F Trapman P. Reproduction numbers for epidemic models with households and other social structures. I. Definition and calculation of $R_{0}$. Math Biosci. (2012) 235(1):85–97. 10.1016/j.mbs.2011.10.009 22085761 PMC3693038

[31] Phillips B Anand M Bauch CT. Spatial early warning signals of social and epidemiological tipping points in a coupled behaviour-disease network. Sci Rep. (2020) 10:1–12. 10.1038/s41598-020-63849-0 32376908 PMC7203335

[32] Carrignon S Bentley RA Silk M Fefferman NH. How social learning shapes the efficacy of preventative health behaviors in an outbreak. PLoS One. (2022) 17:1–17. 10.1371/journal.pone.0262505 PMC8752029 35015794

[33] Fukuda E Kokubo S Tanimoto J Wang Z Hagishima A Ikegaya N. Risk assessment for infectious disease and its impact on voluntary vaccination behavior in social networks. Chaos Solitons Fractals. (2014) 68:1–9. 10.1016/j.chaos.2014.07.004

[34] Wells CR Bauch CT. The impact of personal experiences with infection and vaccination on behaviour-incidence dynamics of seasonal influenza. Epidemics. (2012) 4(3):139–51. 10.1016/j.epidem.2012.06.002 22939311

[35] Easley D Kleinberg J. Cascading behavior in networks. In *Networks, crowds, and markets: reasoning about a highly connected world* (2010). p. 563–609.

[36] Oraby T Thampi V Bauch CT. The influence of social norms on the dynamics of vaccinating behaviour for paediatric infectious diseases. Proc R Soc B Biol Sci. (2014) 281(1780):1–24. 10.1098/rspb.2013.3172 PMC4078885 24523276

[37] Ortega PA Braun DA. Thermodynamics as a theory of decision-making with information-processing costs. Proc R Soc A: Math Phys Eng Sci. (2013) 469(2153):20120683. 10.1098/rspa.2012.0683

[38] Pastor-Satorras R Vespignani A. Epidemic dynamics and endemic states in complex networks. Phys Rev E. (2001) 63(6):066117. 10.1103/PhysRevE.63.066117 11415183

[39] Okuta R Unno Y Nishino D Hido S Loomis C. CuPy: a NumPy-compatible library for NVIDIA GPU calculations. *Proceedings of Workshop on Machine Learning Systems (LearningSys) in The Thirty-first Annual Conference on Neural Information Processing Systems (NIPS)*; Dec 4–9; Long Beach, CA, United States (2017).

[40] NVIDIA, Vingelmann P Fitzek F. Cuda, release 10.2.89 (2020). Available online at: https://developer.nvidia.com/cuda-toolkit (accessed March 1, 2022).

[41] Vynnycky E White R. An introduction to infectious disease modelling. New York: Oxford University Press (2010).

[42] Prentice D Paluck EL. Engineering social change using social norms: lessons from the study of collective action. Curr Opin Psychol. (2020) 35:138–42. 10.1016/j.copsyc.2020.06.012 32746001

