# Supplementary material for: Modeling the effect of observational social learning on parental decision-making for childhood vaccination and diseases spread over household networks
Source: Front Epidemiol. 2024 Jan 12;3:1177752. doi: 10.3389/fepid.2023.1177752 (PMC10910890; doi:10.3389/fepid.2023.1177752)
Supplement: Supplementary file 1 [file Image1.pdf]

Modeling the Effect of Observational Social Learning on Parental Decision-Making for Childhood Vaccination and Diseases Spread over Household Networks

Tamer Oraby*, Andras Balogh
School of Mathematical and Statistical Sciences, The University of Texas Rio Grande Valley, 1201 W. University Dr., Edinburg, Texas, USA,
*Corresponding author: tamer.oraby@utrgv.edu

S. Supplementary Material

SI. Model

In this section, we provide a comprehensive description of the physical and social networks, as well as the dynamics of disease and information spread.

Children's physical network

The first network, denoted as $\mathcal{N}^C$, connects children to households and serves as the platform for disease transmission from child to child. Links between children, representing face-to-face contact opportunities through school, clubs, and communal activities, are assumed to be static; they remain unchanged once established. This assumption is reasonable considering that the modeled outbreaks span only a few weeks. In this network, transmission is exclusively through these connections, with no other routes (such as environmental transmissions) considered. We assume that the network comprises N households, each connected through physical transmission links for every child (agent) in the household. The set of neighbors connected to household i in this network is denoted by $N_P(i)$, with $n_P(i)$ representing the number of these neighbors.

1. **Formation.** A number of children C_i (which can be zero), is randomly assigned to each household i, for $i = 1, \ldots, N$, using a binomial distribution with parameters n_c and p_c. We consider two types of networks:
   (a) **Erdős-Rényi (random) network (ERN)**: Two households, i and j, are physically connected with a probability $p \cdot \sqrt{C_i C_j}$, which is proportional to the geometric mean of the number of children in both households. This probability could be interpreted through an analogy to Newton's law of gravity, a concept that warrants future investigation. A household with zero children will not have physical connections. A regular ERN is a special case where $C_i = 1$ for all i. The parameter p is chosen to be less than $1/n_c$.
   (b) **Barabási-Albert Network (BAN)**: In this network model, the first two households are connected, and each subsequent household is connected iteratively based on the weighted degree distribution of the preceding households. For example, the household j (where $j = 3, 4, \ldots, N$) is connected to one of the households $1, 2, \ldots, j-1$ according to the weighted degree distribution $\dfrac{C_k d(k)}{\sum_{i=1}^{j-1} C_i d(i)}$, where $k = 1, 2, \ldots, j-1$, and $d(k)$ is the degree of household k. A household with zero children will not have physical connections. A regular BAN is a special case where $C_k = 1$ for all k.

See Figure S1, for simulation instances of overlapping children's and parental networks for ERNs and BANs.

2. **Birth process.** We assume that the decision of families to have a new baby (start a new pregnancy) is influenced by the number of children already in the household. Specifically, the probability of a new birth in the household i, assuming that it is not already expecting, is given by the formula
$$\frac{\sigma}{1+\exp(k(C_i - C^*))}.$$
After 280 days, C_i is updated to C_i+1. Here, the parameter σ represents the population-level birth rate, the parameter k measures the sensitivity of the birth probability to the number of children in the household, and C^* is the median of this probability. We have estimated k such that this probability approaches zero as C_i approaches n_C.
The human gestation period is assumed to be 280 days. Following delivery, parents make the decision to vaccinate or not vaccinate their newborns. The initial 280 days of the model's simulation are treated as a burn-in period and thus are not included in the analysis.

3. **Disease transmission within and between households.** Initially, a number of children I_0 are randomly and uniformly selected to be infected. A new infection in say household i, can occur on any given day. This happens due to transmission either within the household or between households, with a probability given by
$$1-(1-\beta_h)^{I(i)} \cdot (1-\beta)^{n_I(i)/C_i}.$$
Here, β represents the probability of infecting a child in another household through the physical network, and β_h is the probability of infecting a sibling within the same household. The formula $I(i) = \sum_{k=1}^{\ell} I(i,k)$ provides the total number of infected siblings in household i, where $I(i,k)$ indicates the number of infected siblings at day/stage k of the incubation period. The expression $n_I(i) = \sum_{j\in N_P(i)} I(j)$ provides the number of infected children connected to household i through the network. We divide $n_I(i)$ by C_i in the infection probability formula to approximate the assumption that children in a household, on average, have the same number of friends. Multiple infections can occure on the same day within the network, and the values of $n_I(i)$ and $I(i)$ are updated daily for all households i, where $i = 1, 2, \ldots, N$.

4. **Disease progression.** The incubation period is modeled using a discretized exponential distribution with a mean length of m_p days and a maximum of ℓ days. An infected child on the jth day after infection either progresses to the end of the incubation period (and recovers) or remains infected into the following day. These outcomes occur with probabilities Q_j and $1-Q_j$, respectively. The probability Q_j is calculated as follows:
$$Q_j = \frac{\int_j^{j+1} f(x)dx}{\int_j^{\infty} f(x)dx}$$
for $j = 0, 1, \ldots, \ell-1$, and $Q_\ell = 1$, where $f(x)$ is the probability density function of the exponential distribution.

Parental social network

The second network, denoted as $\mathcal{N}^P$, overlaps with the children's physical network and includes broader social/information links between parents in different households. In this social social network, parents exchange information and perceive opinions from network neighbors, who may include friends, relatives, colleagues, online connections, and others, beyond just physical neighbors. The set of neighbors connected to household i in this network is represented by $N_S(i)$, with their number being $n_S(i)$. To create an overlapping social network with the children's network, links from the children's physical network are randomly retained with a probability of p_{re}. Additionally, new links are added to any one of the non-connected households updated in the process, with probability p_{ad}.

Observational social learning and its relationship with other models of decision making

In this paper, we introduced the posterior probability as defined in formula (0.1):

$$P_S(i) = \frac{pr_i \cdot \prod_{j\in N_V(i)} q_{j,i} \cdot \prod_{k\in N_N(i)}(1-q_{k,i})}{pr_i \cdot \prod_{j\in N_V(i)} q_{j,i} \cdot \prod_{k\in N_N(i)}(1-q_{k,i}) + (1-pr_i)\cdot \prod_{j\in N_V(i)}(1-q_{j,i}) \cdot \prod_{k\in N_N(i)} q_{k,i}}. \quad (0.1)$$

This formula, which we refer to as the Bayesian aggregation rule in observational social learning, updates the prior probability of vaccination pr_i based on independently collected or perceived information from neighbors in the network. A vaccinator is perceived to hold that opinion with a probability of $q_{i,j}$, and a non-vaccinator is perceived to favor vaccination with a probability of $1-q_{i,j}$.

In nondirectional social learning, characterized by outward uniformity, $q_{j,i} = q_{j,k} =: q_j$ for all $i \neq k$ and for all j.

If $q_j = q$ for all j, then formula (0.1) becomes:

$$P_S(i) = \frac{pr_i \cdot q^{n_V(i)} \cdot (1-q)^{n_N(i)}}{pr_i \cdot q^{n_V(i)} \cdot (1-q)^{n_N(i)} + (1-pr_i)\cdot(1-q)^{n_V(i)} \cdot q^{n_N(i)}} \quad (0.2)$$

for $0 < q < 1$. An uninformative probability of $q = .5$ results in no social influence on the parent, as $P_S(i) = pr_i$.

The model in formula (0.1) can also be rewritten as:

$$P_S(i) = \frac{1}{1+\exp\left(-\Delta_i - \pi_i\right)}, \quad (0.3)$$

where $\pi_i = \text{logit}(pr_i)$ and $\Delta_i = \sum_{j\in N_V(i)} \text{logit}(q_{j,i}) - \sum_{k\in N_N(i)} \text{logit}(q_{k,i})$. Here, $\text{logit}(p) = \log\left(\frac{p}{1-p}\right)$.

Furthermore, by expressing $PS(i)$ in the Boltzmann distribution form, we reveal a bounded rational model supported by physics [1]:

$$P_S\left(i\right) = \frac{\exp\left(.5(\Delta_i + \pi_i)\right)}{\exp\left(.5(\Delta_i + \pi_i)\right) + \exp\left(.5(\bar{\Delta}_i + \bar{\pi}_i)\right)}$$

with a temperature of $\tau = .5$. Here, the reward for vaccination is given by $\pi_i = \alpha_i I - \gamma_i A = -\bar{\pi}_i$, while $\Delta_i = \sum_{j \in N_V(i)} \text{logit}\,(q_{j,i}) - \sum_{k \in N_N(i)} \text{logit}\,(q_{k,i}) = -\bar{\Delta}_i$ represents the induced social pressure to vaccinate.

The DeGroot model of selection [2], is reflected in the term Δ_i if each neighbor $j \in N_S(i)$ is assigned a weight of $\text{logit}(q_{j,i})$, using discrete opinions valued as $+1$ for vaccination and -1 for no vaccination. This situates a stochastic DeGroot model of selection as a special case within the framework of the Bayesian aggregation rule in equation (0.1).

Similarly, when $q_i = q$ and $pr_i = pr$ for all i, as in the model of equation (0.2), the model can be reformulated as

$$P_S(i) = \frac{1}{1 + \exp\left(-\delta_i \dfrac{n_V(i) - n_N(i)}{n_S(i)} - \pi\right)}, \tag{0.4}$$

where $\pi = \text{logit}(pr)$ and $\delta_i = n_S(i)\,\text{logit}(q)$ signifies the degree of injunctive social norm practiced by household i. Refer to [3, 4, 5] for the case where $\delta_i =: \delta$ for all i, leading to the formula

$$P_S(i) = \frac{1}{1 + \exp\left(-\delta(2\frac{n_V(i)}{n_S(i)} - 1) - \pi\right)}, \tag{0.5}$$

which aligns with the voting model of selection, using $G := 2\frac{n_V(i)}{n_S(i)} - 1$ to decide the winning strategy when $G > 0$ and the losing strategy when $G < 0$. Thus, this latter model is also a special case of (0.1) in scenarios of homogeneous observational learning. However, since δ is nonnegative, formula (0.5) only reveals human behavior when $q \geq 0.5$, making it less comprehensive than the model in (0.1).

SII. Parameter Values

The model simulation is performed many times with different sets of selected parameter values. A parameterization of the model is detailed in Table S1. These parameters were determined based on literature review, calibration, and guesstimation. The unit of time used in the model is measured in days.

Table S1: Model parameters, their description and base values.

Parameter	Description	Base values
N	Number of households (nodes)	$100,000$
σ	Birth rate	.005
n_c	Maximum number of children per household	7
p_c	Initial probability of having a child in a household	.4
k	Sensitivity of pregnancy probability to number of children	2.5
C^*	Median number of children in probability of pregnancy	2
p	Physical connection probability	.00013
p_{re}	Probability of retaining a physical connection to form the social network	.6
p_{ad}	Probability of adding a connection to the social network	.0004
I_0	Initial number of infected children	10
β	Human to human transmission probability through network [3]	.06
h	Human to human factor of transmission probability within household	1.5
m_p, i_p	Mean and maximum length of infectious period	11, 16 days
NV_0	Initial number of all time non-vaccinators	$5\% \cdot N$
MS_0	Initial number of mover-stayer parents	$N - NV_0$
P_S	Probability to vaccinate with social learning	–
e	Vaccine efficacy	.95
p_{adv}	Probability of vaccine adverse event	$.0001 - .01$
α	Degree of relevance of infectiousness to the decision to vaccinate	.001
γ	Degree of relevance of vaccine adverse events to the decision to vaccinate	.01
q	Probability to give a correct perception of vaccination	$0 - 1$
δ	Injunctive social norm	$.025 - .225$
ρ	Probability to get access to vaccination	$.001 - .01$

SIII. Supplementary Figures

SIV. Estimating the Basic Reproduction Number R_0

The basic reproduction number is defined as the average number of secondary infections caused by an index case introduced into a completely susceptible population until its recovery. To calculate the basic reproduction number, as defined for the disease process over a network of households $\mathcal{N}^C$, we implement algorithm 1. This algorithm is based on running the disease process multiple times for an index case, which is uniformly selected from the network. The expected number of cases caused by the index case is then estimated using the mean of binomial distributions. The probabilities for these distributions are determined using Bayes' theorem.

In particular, $P_i(t)$ and $P_j(t)$ represent the probabilities that the index case is the cause of infection of a new case within the same household i and among neighboring households j, respectively. Consequently, the mean numbers of infections among $IC_i(t)$ and $IC_j(t)$ that are attributed to the index case are calculated as $IC_i(t)P_i(t)$ and $IC_j(t)P_j(t)$, respectively.

SV. Coding of Simulation.

Our numerical simulations utilize the Python library CuPy [6], which is compatible with NumPy, and are accelerated with NVIDIA CUDA [7] for parallel calculations on Graphical Processing Units

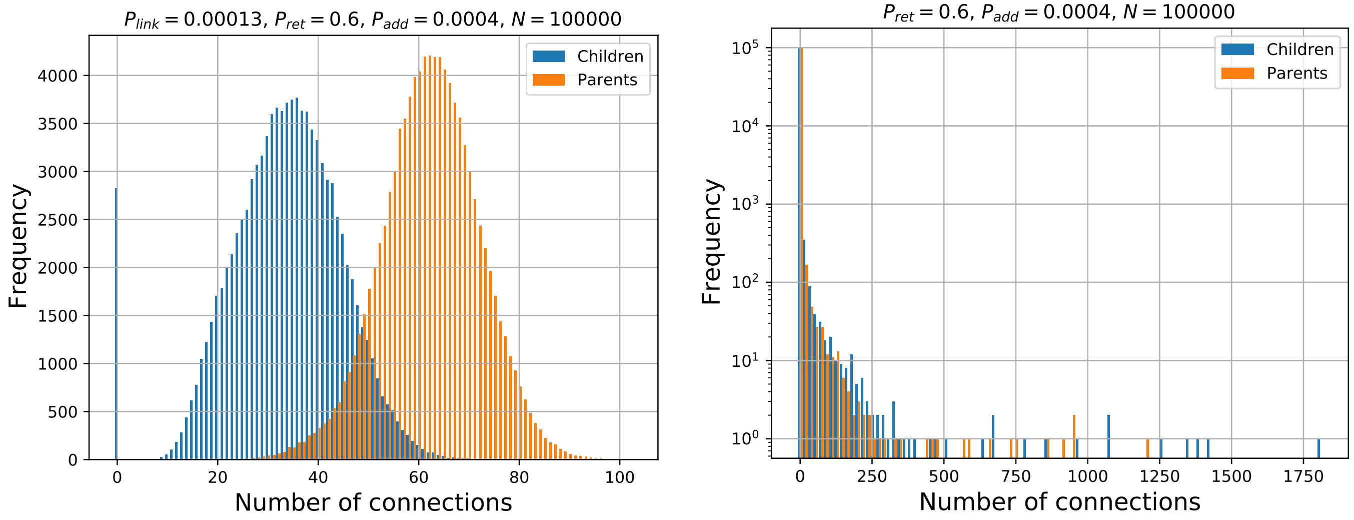

(a) (b)

Figure S1: **Degree histogram for a simulation of Erdős-Rényi (random) network model (ERN) in (a) and Barabási-Albert network model (BAN) in (b).**

(GPUs). Most of the calculations were performed on a GPU workstation with 8 NVIDIA Tesla (Kepler) K80 GPU cards, each having 2496 CUDA Cores and 12GB memory. Additionally, the code was also tested on a GPU cluster with 8 NVIDIA A100 SXM GPU cards, each boasting 6912 CUDA cores and 80GB memory. GPU-accelerated calculations provide, on average, a speed-up of $10 - 20$ times over CPU calculations. In the case of reduction operations, such as summation over large arrays – which our code often uses – the speed up can often reach $100\times$ through the support of more performant backends on NVIDIA GPUs.

We introduce a sparse storage format for the adjacency matrices of the networks, in which we only record the positions of the nonzero elements in the upper triangular part using a single index number (refer to Figure S6). In this format, k denotes the index of the entries in the upper triangular part, arranged in column-major order, while (i, j) corresponds to the respective row and column indices the following relationships hold between these indices:

$$k = i + \frac{(j-2)(j-1)}{2}, \tag{0.6}$$

$$j = \left\lfloor \frac{3 + \sqrt{8k - 7}}{2} \right\rfloor, \tag{0.7}$$

$$i = k - \frac{(j-2)(j-1)}{2}. \tag{0.8}$$

Indexing formulas (0.6)–(0.8) provide a fast and efficient storage and calculations through parallel implementations on the GPUs.

Eight GPU cards were utilized to distribute the 100 runs of stochastic simulations, with each graphics card executing one network simulation at a time. Although it is possible to run the epidemic process process immediately after generating a network, we typically conduct these two

processes separately. Following network generation, the networks are saved to a hard drive. Then, at the start of the epidemic process, these networks are loaded into memory from the hard drive. Timing information on NVIDIA A100 SXM GPUs:

- Average generation time of one network, including the savings of the networks to hard drive:
  - Erdős-Rényi Network (ERN): 4 seconds of wall clock time.
  - Barabási-Albert Network (BAN): 62 seconds of wall clock time. This network generation involves more serial steps than the ERN, as households are added to the network one by one.
- Average epidemic run time over one network:
  - Erdős-Rényi Network (ERN): 7 seconds of wall clock time.
  - Barabási-Albert Network (BAN): 5 seconds of wall clock time. The shorter time is attributed to the smaller size of the epidemics in this case compared to those in the ERNs.

References

[1] P. A. Ortega and D. A. Braun, "Thermodynamics as a theory of decision-making with information-processing costs," *Proceedings of the Royal Society A: Mathematical, Physical and Engineering Sciences*, 2013.

[2] A. Namatame and S.-c. Chen, *Agent-based modeling and network dynamics.* Oxford University Press, 2016.

[3] B. Phillips, M. Anand, and C. T. Bauch, "Spatial early warning signals of social and epidemiological tipping points in a coupled behaviour-disease network," *Scientific Reports*, vol. 10, pp. 1–12, dec 2020.

[4] T. Oraby, V. Thampi, and C. T. Bauch, "The influence of social norms on the dynamics of vaccinating behaviour for paediatric infectious diseases," *Proceedings of the Royal Society B: Biological Sciences*, vol. 281, no. 1780, pp. 1–24, 2014.

[5] T. Oraby and C. Bauch, "Bounded rationality alters the dynamics of paediatric immunization acceptance," *Scientific Reports*, vol. 5, no. 10724, 2015.

[6] R. Okuta, Y. Unno, D. Nishino, S. Hido, and C. Loomis, "Cupy: A numpy-compatible library for nvidia gpu calculations," in *Proceedings of Workshop on Machine Learning Systems (LearningSys) in The Thirty-first Annual Conference on Neural Information Processing Systems (NIPS)*, 2017.

[7] NVIDIA, P. Vingelmann, and F. Fitzek, "Cuda, release: 10.2.89," 2020.

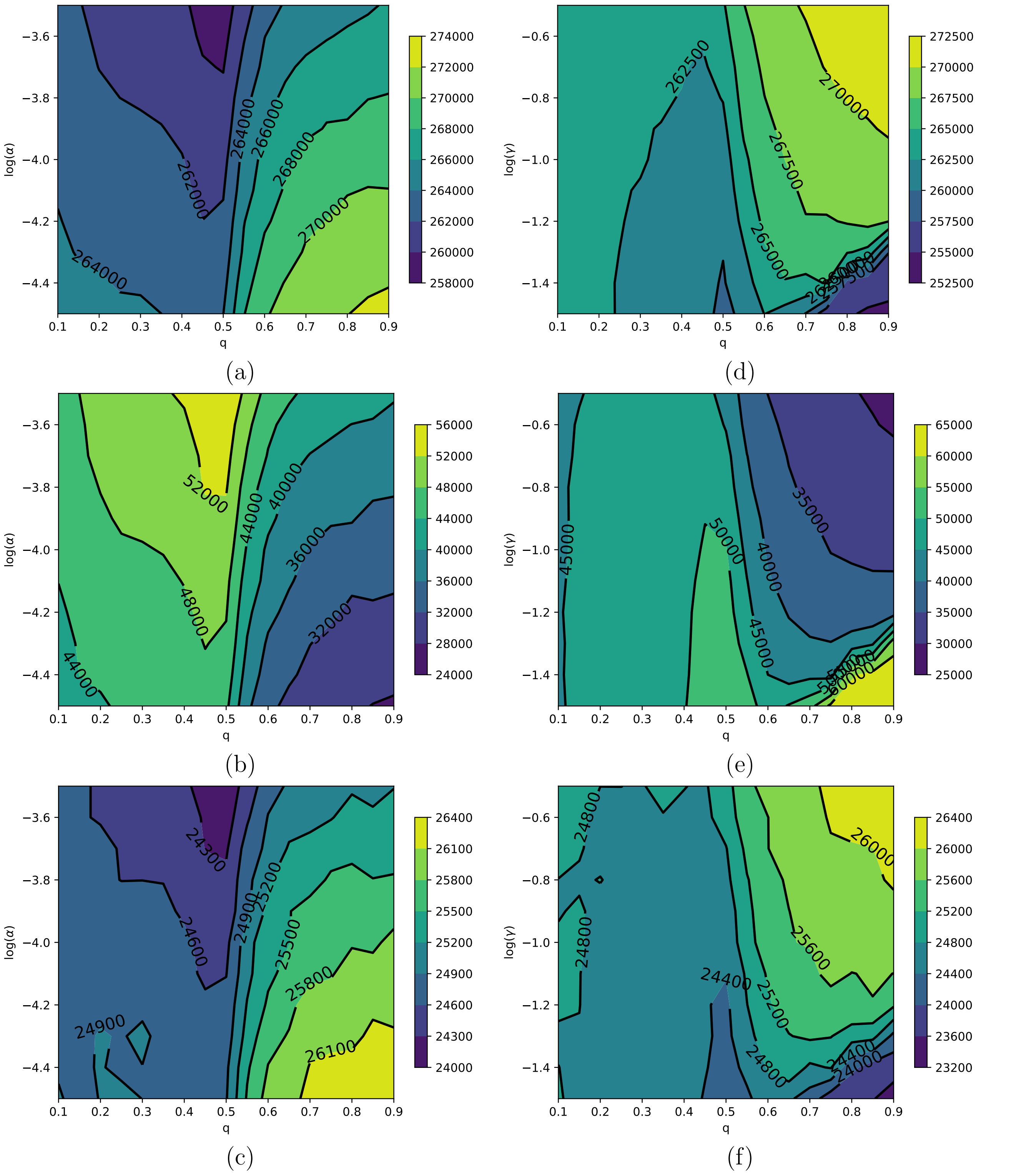

Figure S2: **Parameter planes of α against q and γ against q for sizes of the epidemic, the total number of vaccinated children, and the peak of the epidemic on Erdős-Rényi (random) network model (ERN).** In all simulations, the median value of the simulations are used to plot the parameter planes that are performed at $P_{adv} = .001$ and $\rho = .01$.

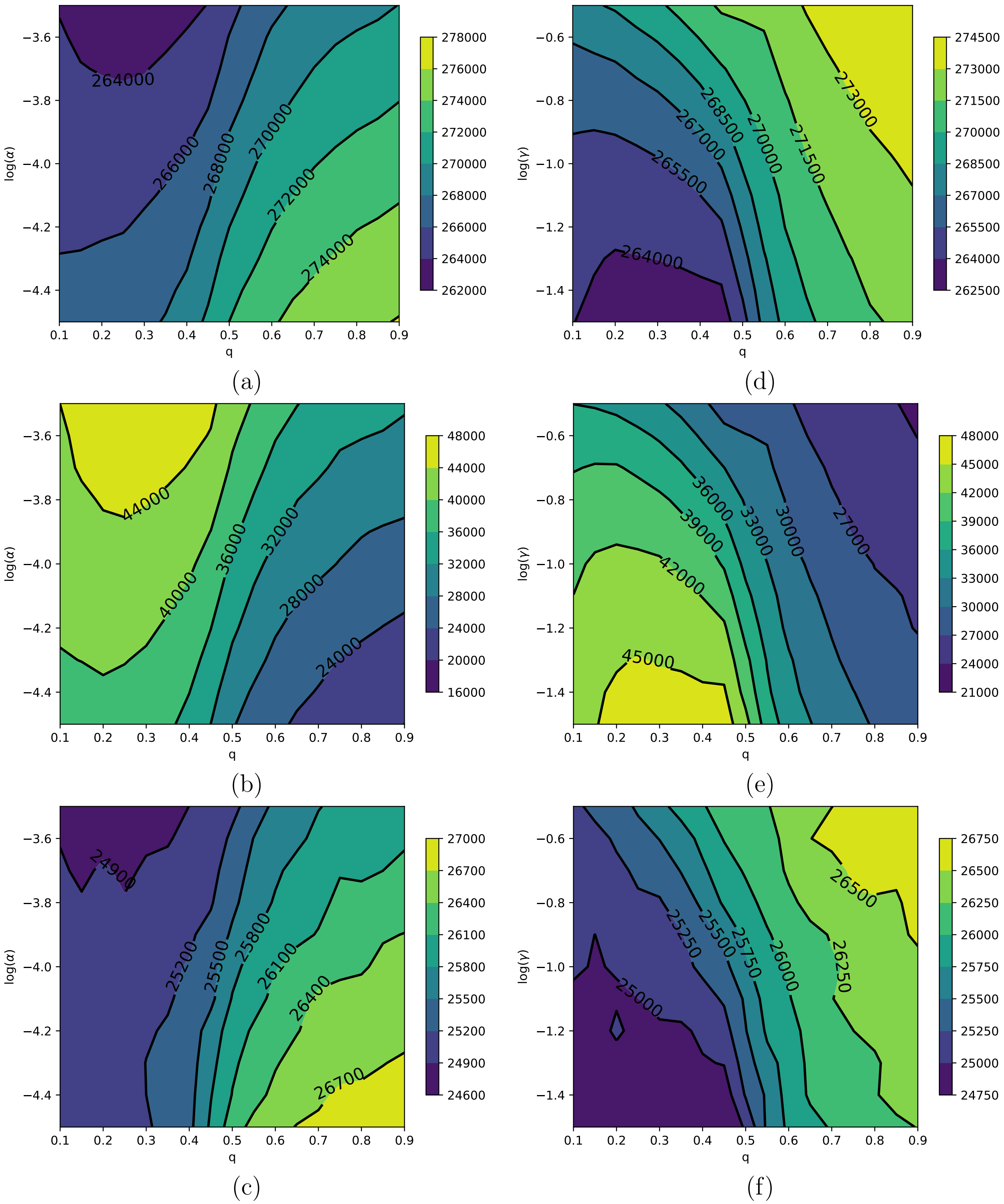

Figure S3: **Parameter planes of α against q and γ against q for sizes of the epidemic, the total number of vaccinated children, and the peak of the epidemic on Erdős-Rényi (random) network model (ERN).** In all simulations, the median value of the simulations are used to plot the parameter planes that are performed at $P_{adv} = .01$ and $\rho = .01$.

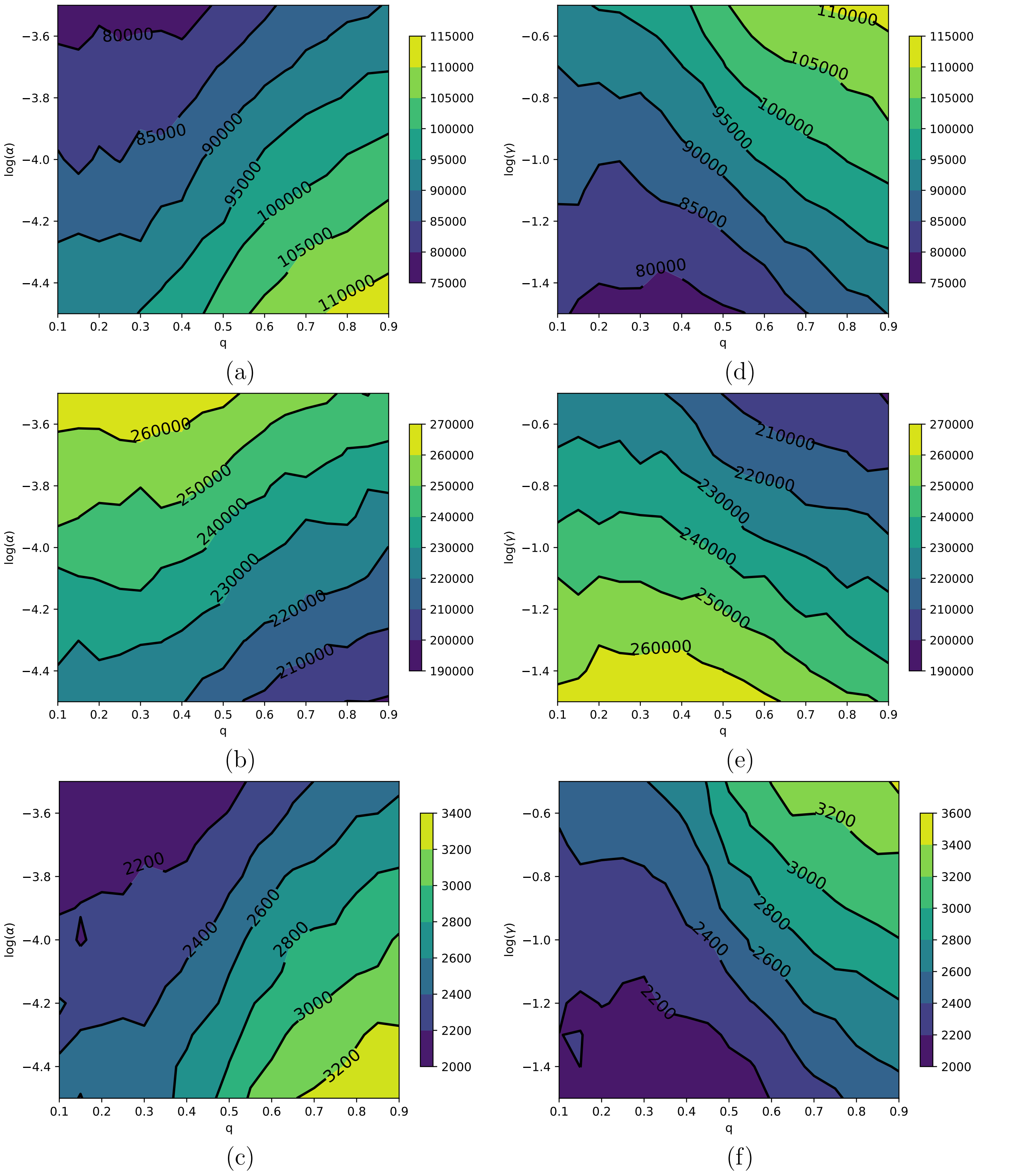

Figure S4: **Parameter planes of α against q and γ against q for sizes of the epidemic, the total number of vaccinated children, and the peak of the epidemic on Barabási-Albert network model (BAN).** In all simulations, the median value of the simulations are used to plot the parameter planes that are performed at $P_{adv} = .001$ and $\rho = .01$.

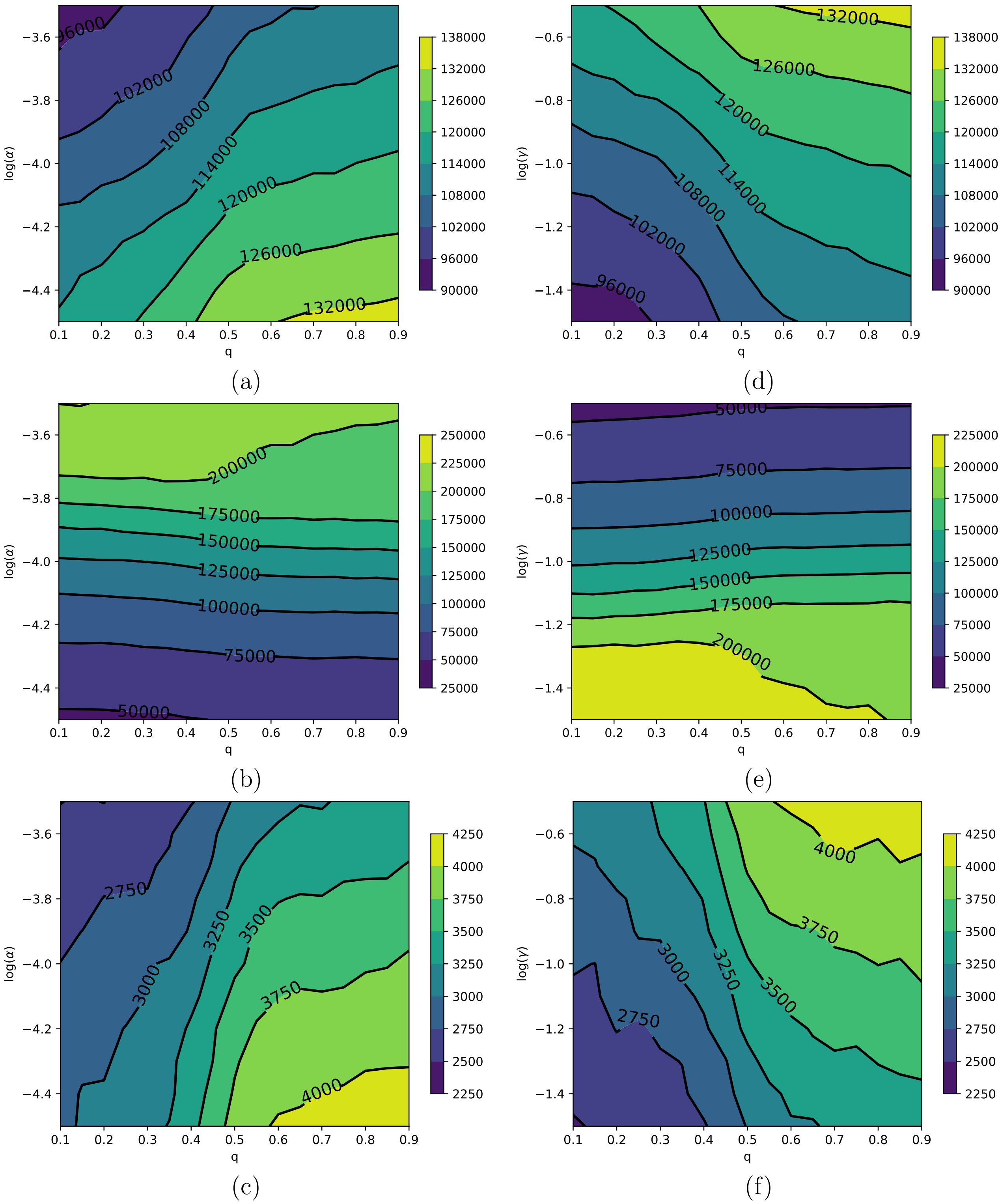

Figure S5: **Parameter planes of α against q and γ against q for sizes of the epidemic, the total number of vaccinated children, and the peak of the epidemic on Barabási-Albert network model (BAN).** In all simulations, the median value of the simulations are used to plot the parameter planes that are performed at $P_{adv} = .01$ and $\rho = .01$.

Algorithm 1 To estimate the basic reproduction number using simulations of disease processes on networks.

Input: number of simulation runs of the disease process L, number of simulated networks W, inputs for process in table S1

Output: R_0

begin

for $w = 1, 2, \ldots, W$

▷ Generate a network $\mathcal{N}_w^C$ of N households and number of children in households $\{C_i(0) : i = 1, \ldots, N\}$. Also, generate the starting stand of households about vaccination.

for $i = 1, 2, \ldots, N$, such that $C_i(0) > 0$

for $\ell = 1, 2, \ldots, L$

▷ Select one child in household i to be the index case

▷ Run the disease process until the index case is recovered, say until day T, which will change from one run to another.

▷ Record the number of new cases in the household j, $IC_j(t)$ and the total number of prevalent children in the household j less the index case, $I_j(t)$, for all $j \in \{i\} \cup N_P(i)$. Record the total number of children who are disease prevalent in the outer neighbors k of the household j, $I_{j,k}(t)$, for $t = 1, 2, \ldots, T$ for all $j \in N_P(i)$, and $k \in N_P(j) - \{i\}$

▷ Calculate:

The probability that an infection occurred in the house i due to the index case in household i

$$P_i(t) = \frac{h\beta}{h\beta + (1 - (1 - h\beta)^{I_i(t)}) + \sum_{j \in N_P(i)} (1 - (1 - \beta)^{I_j(t)/C_j(t)})}$$

and the probability that an infection in the household j occurred due to the index case in household i

$$P_j(t) = \frac{\beta}{\beta + (1 - (1 - \beta)^{I_i(t)}) + (1 - (1 - h\beta)^{I_j(t)}) + \sum_{k \in N_P(j) - \{i\}} (1 - (1 - \beta)^{I_{j,k}(t)/C_{j,k}(t)})}$$

where $C_{j,k}(t)$ are the number of children in the outer neighbors k of the household j.

▷ Calculate:

$$R_0(w, i, \ell) = \sum_{t=1}^{T} \sum_{j \in \{i\} \cup N_P(i)} IC_j(t) P_j(t)$$

end

▷ Return $R_0(w, i) = \frac{1}{L} \sum_{\ell=1}^{L} R_0(w, i, \ell)$

end

▷ Return $R_0(w) = \frac{1}{N} \sum_{i=1}^{N} R_0(w, i)$

end

▷ Return $R_0 = \frac{1}{W} \sum_{w=1}^{W} R_0(w)$

end

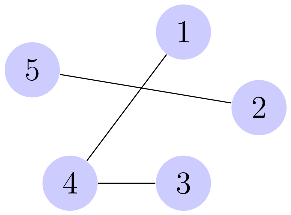

$$A = \begin{bmatrix} 0 & 0 & 0 & 1 & 0 \\ 0 & 0 & 0 & 0 & 1 \\ 0 & 0 & 0 & 1 & 0 \\ 1 & 0 & 1 & 0 & 0 \\ 0 & 1 & 0 & 0 & 0 \end{bmatrix} \qquad \begin{bmatrix} . & 1 & 2 & 4 & 7 \\ . & . & 3 & 5 & 8 \\ . & . & . & 6 & 9 \\ . & . & . & . & 10 \\ . & . & . & . & . \end{bmatrix}$$

Figure S6: **One-index representation of network models.**
